# A High-throughput, Robotic System for Analysis of Aerosol Sampling Filters

**DOI:** 10.4209/aaqr.210037

**Published:** 2021-11

**Authors:** Christian L'Orange, Gabe Neymark, Ellison Carter, John Volckens

**Affiliations:** 1 Mechanical Engineering, Colorado State University, Fort Collins CO, USA; 2 Civil and Environmental Engineering, Colorado State University, Fort Collins CO, USA

**Keywords:** Particulate matter, Gravimetric analysis, Air pollution, Black carbon, PM_2.5_

## Abstract

The determination of accumulated mass on filter-based aerosol samples is the basis for many forms of scientific research and regulatory monitoring of air quality. However, gravimetric analysis of air sampling filters is tedious, time-intensive, and prone to human error. This work describes the development of an **A**utomated A**ir** Ana**l**ys**i**s **F**acili**t**y (AIRLIFT) for high-throughput gravimetric mass and optical black carbon measurements of filter-based aerosol samples. The AIRLIFT consists of a sealed environmental enclosure, a 6-axis articulating robotic arm, a programmable control system, a filter weighing apparatus, and an optical system for the determination of aerosol black carbon via light attenuation. The system actively monitors microbalance stability and chamber relative humidity. Digital imaging and QR code scanning support sample tracking and data logging. Performance metrics for temperature and humidity control and weight stability were found to meet or exceed minimum requirements set forth by the US Environmental Protection Agency. The AIRLIFT is capable of analyzing approximately 260 filters per day while reducing the required personnel time by a factor of ~4.

## 1 INTRODUCTION

Exposure to airborne particulate matter (PM) is the leading environmental risk factor for premature disease and death on the planet (Institute for Health Metrics and Evaluation, 2019). The gold-standard method for determination of PM mass concentrations is gravimetric analysis of air sampling filters. The United States Environmental Protection Agency (U.S. EPA) describes gravimetric analysis as the sole *Federal Reference Method* for the determination of PM_2.5_ and PM_10_ concentrations (particles with aerodynamic diameters ≤ 2.5 μm and 10 μm, respectively) in air; alternative methods (such as light-scattering, beta attenuation, and the tapered-element oscillating microbalance) are deemed *Federal Equivalence Methods* because they must demonstrate equivalence to gravimetric filter analysis prior to their acceptance and use (Baldwin, 1987; Code of Federal Regulations, 2006). Other standard-setting bodies (e.g., US Occupational Safety and Health Administration (OSHA, 2003), the European Union (CSN-EN, 2014)) also rely heavily on gravimetric methods for outdoor and workplace exposure limits. Unfortunately, gravimetric filter analysis is time-consuming, tedious, and prone to bias/imprecision unless strict quality control procedures are employed (Code of Federal Regulations, 1987, 2006; U.S. EPA, 1998).

Gravimetric analysis requires quantifying the mass of PM accumulated on an air sampling filter (i.e., weighing the filter on an analytic microbalance before and after a timed air sample is collected at a pre-determined flow rate). Precise, unbiased measurements are needed because the differential mass of accumulated PM is often small compared to the total filter mass. The resolution and precision required of a balance used for gravimetric analysis depends on study specific parameters; however, the microbalances used for aerosol filter analysis often have resolutions of 1 μg or even 0.1 μg. At this mass resolution, static electricity and/or slight variations in environmental conditions can bias the measurement. Regulatory agencies such as the U.S. EPA, among others, have established requirements for gravimetric analysis. Slight differences in procedures and guidelines exist between different regulatory agencies; however, many published procedures include similar approaches and performance limits, such as control of ambient temperature and humidity (and filter equilibration at these conditions), electrostatic discharge of the filter prior to weighing, and rules for filter handling and storage (Code of Federal Regulations, 1987, 2006). These regulatory standards are intended to help ensure precise and repeatable gravimetric measurements. Method imprecision, either though variability in repeated measures or changes in sample mass due to environmental influences, can worsen the limit of detection (LOD) of the method, thereby diminishing the usefulness of the measurements.

The challenge of making precise and unbiased gravimetric filter measurements is compounded by the tedious, labor-intensive nature of the measurement. Human errors are common and include failure to maintain steady and appropriate environmental conditions, failure to wait until the balance has stabilized before recording a reading, and transcription mistakes (when manually recording readings) (Presler-Jur et al., 2016). The repetitive nature of filter weighing is monotonous; thus, the likelihood of human error increases with sample size as individuals tend to rush the weighing process or become negligent to proper filter handling procedures. The weighing of filters is also time-intensive. Each measurement of a gravimetric filter can take several minutes. Filters are typically weighed multiple times (three measurement replicates is common) to ensure quality control and repeatability of the measurements (EPA guidelines require filter measurements to agree within 15 μg (U.S. EPA, 1998)). These repeated measures are critical to ensure measurement quality but result in the weighing process taking a substantial amount of time. A trained technician in our laboratory, following the EPA protocol, can manually weigh only 10–15 filters per hour. This filter weighing rate is similar to rates published by other groups (Presler-Jur et al., 2016). Accurate filter weighing is not only time-intensive from a personnel standpoint, but also requires filters to sit for many hours prior to being weighed in order to allow them to reach equilibrium with their surroundings. This equilibration time requires establishing dedicated areas where filters can sit for hours or days prior to being weighed.

Robotic, automated gravimetric analysis systems can decrease human error and reduce personnel burden while increasing analysis throughput. Robotic systems can also achieve the environmental conditions and precision required to comply with required regulatory gravimetric analysis protocols (Ogden et al., 1996; Presler-Jur et al., 2016). Several automated weighing systems are commercially available (Mettler Toledo, 2021; MTL Corp., 2019); however, these systems are difficult to customize, have limited options for integrating additional measurements, and are often cost-prohibitive. The objective of this work was to develop a more cost-effective, high-throughput, automated filter analysis system that was compliant with standard gravimetric filter analysis requirements (this work focuses on U.S. EPA guidelines) and configurable to include additional filter analysis techniques such as those that support the determination of the chemical composition of particles.

## 2 METHODS

**Fig. 1.**
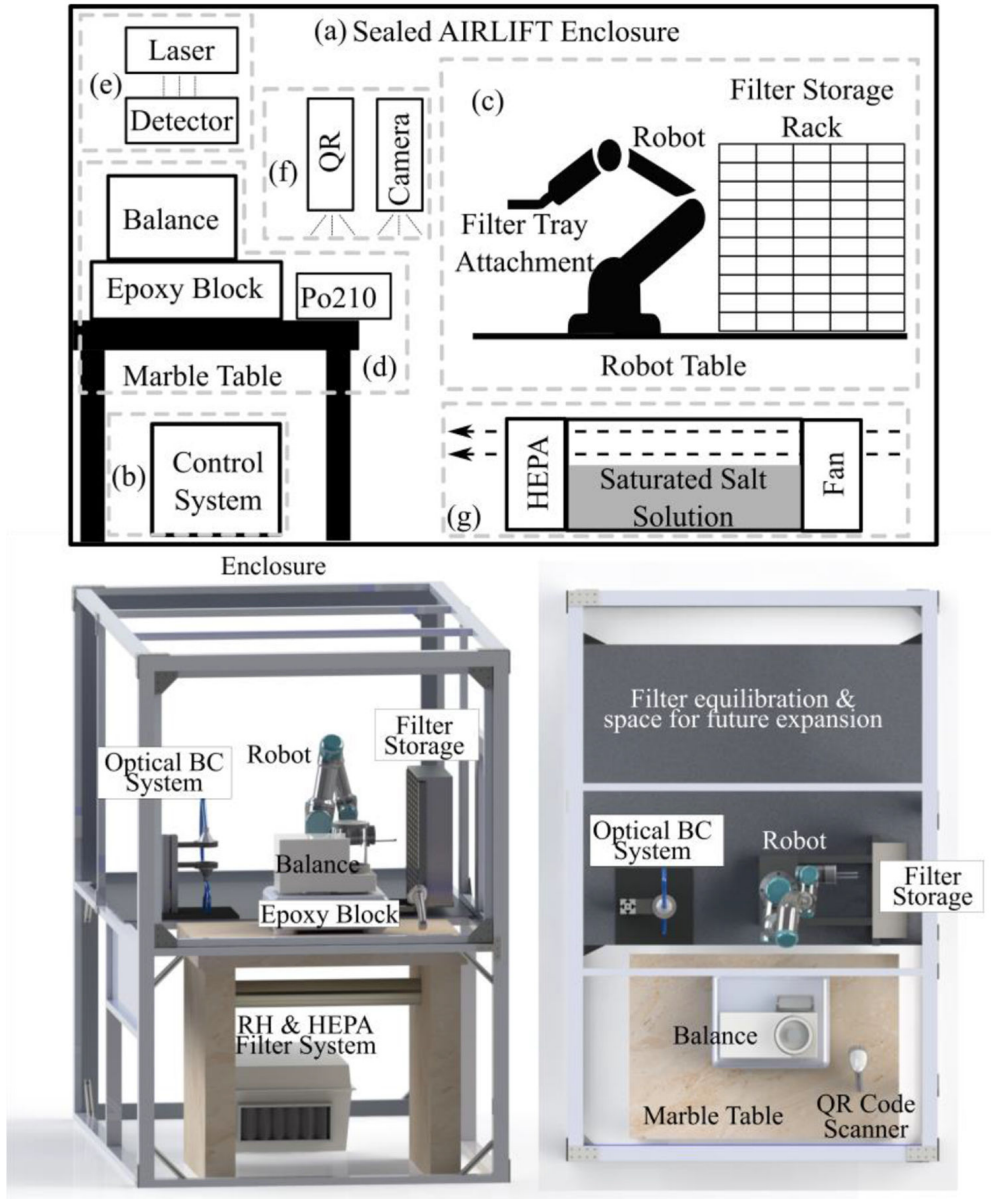
(Top) Schematic view of the AIRLIFT system. Lower-case letters and dotted lines designate subsystems. (Bottom) Side and overhead views of solid model detailing the position of major components within the AIRLIFT system.

We developed the **A**utomated A**ir** Ana**l**ys**i**s **F**acili**t**y (AIRLIFT) to support high-throughput, high-quality analysis of PM sampling filters. The AIRLIFT was designed to support gravimetric and other non-destructive filter analysis techniques, such as optically-measured black carbon. The goals of the AIRLIFT were to (1) increase sample throughput, (2) achieve good analytic figures of merit (bias, precision, and limit of detection), (3) reduce personnel burden, and (4) serve as a platform for multiplexed analysis of air sampling filters. We evaluated the AIRLIFT design against EPA minimum performance requirements for gravimetric analysis (Code of Federal Regulations, 1987, 2006) and against established optical methods for fine particulate black carbon (Ahmed et al., 2009; Hansen et al., 1984). The AIRLIFT includes seven subsystems, as discussed below and depicted in Fig. 1: (a) an enclosure; (b) a data acquisition/control system; (c) a robotic filter management system; (d) a weighing apparatus; (3) and an optical black carbon analysis system; (f) a sample tracking system; and (g) a system for active environmental control. Photographs of the AIRLIFT can be found in Fig. S1 in the supplemental material.

### 2.1 Enclosure

The AIRLIFT includes a sealed enclosure to allow for control of ambient humidity, filtration of air within the enclosure, as well as reduction of vibration and indoor air currents that can affect measurement precision. The enclosure, measuring 1.9 m × 1.2 m × 1.7 m, is constructed from extruded "t-frame" aluminum (80/20 Inc., Columbia City, IN, USA) and 0.25-inch thick acrylic wall panels (Fort Collins Plastic, Fort Collins, CO, USA). The enclosure has doors and removable “access panels” with sealing rubber gaskets for sample loading/unloading and system maintenance. These doors are kept closed while weighing filters to prevent air currents or drafts that might impact the reading of the microbalance.

The AIRLIFT enclosure is 2–3 times larger than would be required just for the physical weighing of filters. The increased chamber volume supports additional space to equilibrate filters before weighing (typically 24 hours) and facilitates future expansion to include other non-destructive filter analysis techniques. Filter equilibration is the process of allowing a filter to reach the same temperature and humidity as the microbalance and the environment where weighing is going to occur. Failing to properly equilibrate filters can result in measurement bias due to microbalance instability and filter mass loss/gain due to moisture loss/uptake (Mettler Toledo, 2015). Proper measurement practices require that filters equilibrate to the same temperature and relative humidity as the balance for at least 24 hours before weighing (Code of Federal Regulations, 1987, 2006). Because the enclosure can accommodate hundreds of unused filters, new, fully-equilibrated filters are ready to be weighed and placed into service as needed. The reduced volume of the AIRLIFT enclosure (relative to a weighing room facility) allows for higher air-exchange rates to be achieved, in addition to a reduced air mass to be conditioned when doors are opened to load or unload filter media.

### 2.2 Data Acquisition and Control System

Data acquisition and control of the AIRLIFT are provided by National Instrument hardware (cRIO-9066, National Instruments, Austin TX USA) and a custom “state machine” LabVIEW program. The control flow logic of a state machine program allows for easy development and debugging.

Two processes operate in parallel to control the operation of the AIRLIFT. One process monitors temperature and humidity levels in the AIRLIFT to ensure the system is within the required ranges. These data are logged to system memory so that AIRLIFT operators can review historical temperature and humidity trends on the graphical user interface prior to each set of analyses. The second process controls the handling and weighing of filters (see below for details). All data collected by the AIRLIFT are backed up to a remote server for redundancy and data integrity.

An automated weighing system, such as the AIRLIFT, can eliminate many of the human errors associated with manual weighing, but also eliminates human review of each individual data point. As a result, there is a risk that any errors or quality control issues that persist in the automated system will not be identified. Several steps have been included in the AIRLIFT data acquisition process to mitigate this risk. Summary statistics are generated for each filter, including measurement range (maximum weight minus minimum weight) and standard deviation. The summary statistics allow an operator to determine if additional measurements should be collected before releasing pre-weighed filters (filters that have not yet been used to collect a sample) for use or post-weighed filter (filters after they have been used) data for analysis. Quality control metrics, such as environmental conditions, background PM levels, and reference weight measurements, are recorded and presented through a graphical user interface. Examples of these quality control indicators can be found in the supplemental information.

### 2.3 Robotic Filter Handling and Storage

**Fig. 2.**
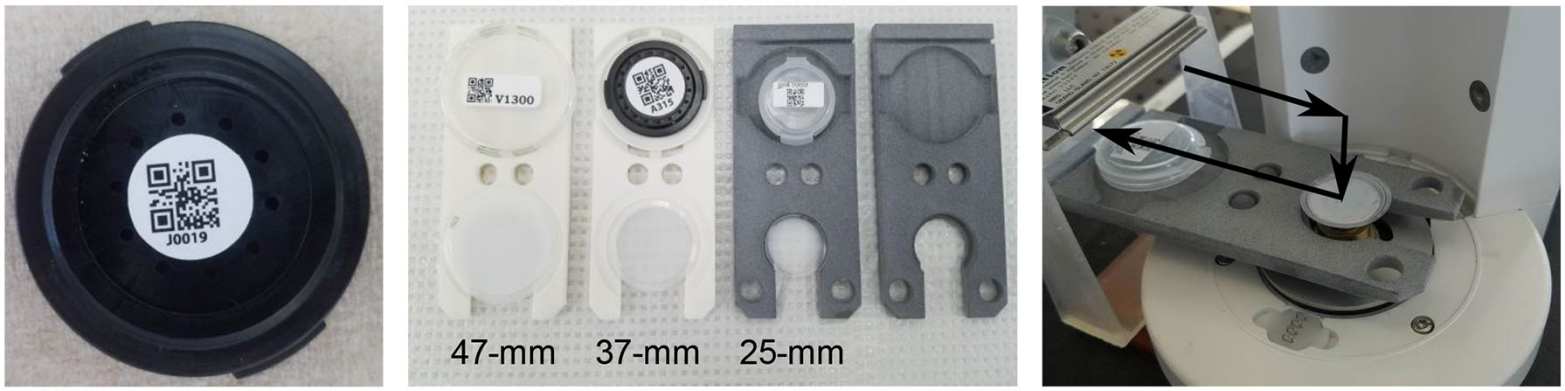
(Left) Example of a filter cartridge labeled with a QR code. The unique identification number allows filter tracking during pre-weighing, sampling, and post-weighing. (Middle) Example filter trays. (Right) The path of motion used to place filters on the weighing pan.

The system to store and handle filters in the AIRLIFT has three primary elements: custom-designed filter transport trays, a custom-designed filter storage rack, and a six-axis robotic arm (UR3, Universal Robots, Odense Denmark). Each filter is loaded into a 3D-printed transport tray. Each tray holds one filter along with a sampling cartridge or storage dish marked with the filter’s unique ID (Fig. 2-middle). These unique filter IDs are used to identify and track filters both during weighing and throughout sampling campaigns. The filter rests on a small ledge that runs approximately 80% of the filter's circumference—leaving the bottom and one side of the filter exposed. Separate tray designs exist for three common filter diameters, 47-mm, 37-mm, and 25-mm.

Each tray is placed in the storage rack to await pickup by the robot arm. The storage rack consists of a series of slots configured in a rectangular grid. Our current storage rack was designed to hold 100 filters (though we estimate capacity could be increased to ~500 filters without substantial changes to the enclosure or AIRLIFT system).

The UR3 robot arm, which moves filters/trays throughout the AIRLIFT, has a positional accuracy of 1 mm, can integrate with LabVIEW, and is designed to accept custom tool attachments. The head of the AIRLIFT robot is equipped with two horizontal rails, similar to forklift arms. These rails allow the robot to pick up and move the filter trays. The tool is attached to the end of the robot head with neodymium magnets to enable the tool to break-away in the event the robot encounters resistance or is out of alignment. Filters rest on a small ledge in the tray that runs approximately 4/5ths of the filter's circumference. The design allows the bottom and one side of the filter to be exposed. The tray is then lowered around the weighing pan, which allows the filter to come to rest on the weighing pan as the tray lowers. The tray then pulls away from the balance, leaving the filter in place, Fig. 2-right. The robot moves in reverse order to pick up a filter from the balance. The UR3 arm was selected for its compact size and easy to access control interface. Details of the major system elements, including cost, are presented in the supplemental information.

### 2.4 Gravimetric Measurements

Filters are weighed on an analytic microbalance (Mettler Toledo XS3DU, Columbus, OH, USA) with a resolution of 1 μg and reported repeatability of 1 μg. Minor modifications were made to the microbalance, including a custom draft shield and a modified weighing pan. The standard weighing pan had an outer diameter of 27 mm; we developed a modified pan of similar shape but with a 20-mm outer diameter to accommodate smaller filters. Accuracy of the balance was verified after modifying the weighing pan by measuring a series of calibration weights. The microbalance is situated on a marble table and epoxy block within the enclosure to minimize the impact of external vibrations (Kuo et al., 2015; Lawless and Rodes, 1999). Static charge is removed from filters using a polonium 210 radiation source (2U500, NRD, Grand Island, NY, USA) that is held facing downward. The UR3 brings each filter to within 10 mm of the radiation source and holds the filter in place for a period of 30 seconds prior to weighing.

**Fig. 3.**
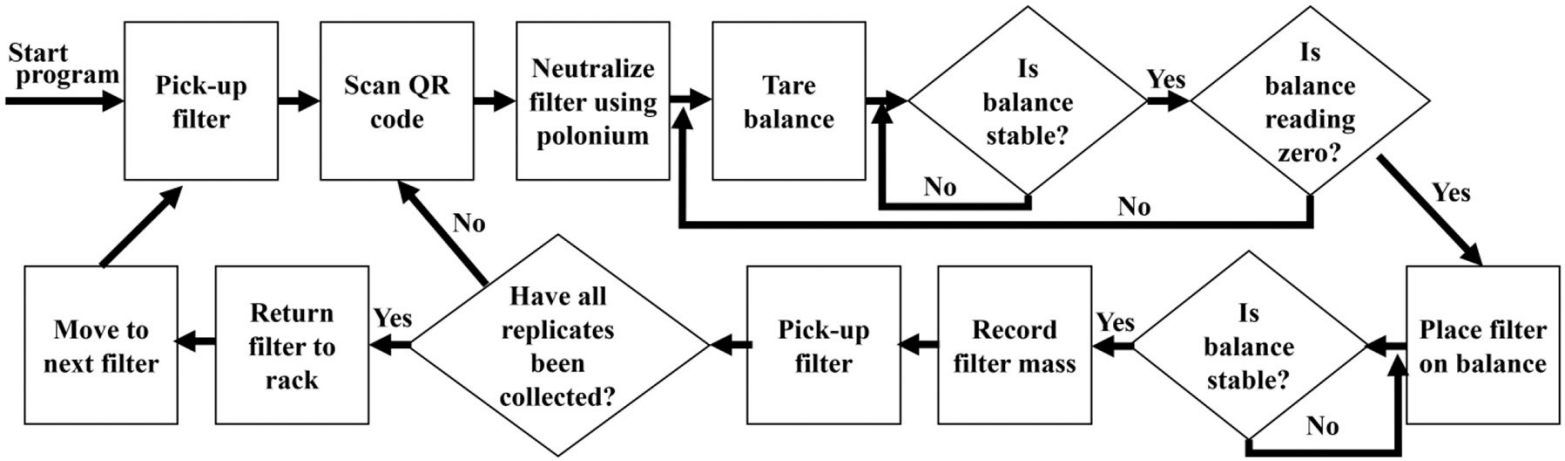
Schematic of filter weighing workflow inside the AIRLIFT.

The AIRLIFT uses RS-232 serial protocols to communicate between the LabVIEW program and the microbalance. The control system can receive output data from the balance and also input zero/tare commands to the balance. The XS3DU microbalance has internal protocols to determine if a current reading is "stable" (Mettler Toledo, 2015). The control program can query the current stability status from the microbalance and either wait, if the balance is not stable, or move on to the program's next phase. Once stable, the AIRLIFT records the current mass reading. A summary workflow of the AIRLIFT weighing process is presented in Fig. 3. The user can specify the number of replicates to collect for each sample. The system follows the same procedure for each replicate: scan QR code, neutralize static, tare balance and ensure stability, weigh filter. Once all replicates for a given filter are completed, the filter is returned to the filter storage rack and the UR3 proceeds to the next filter.

### 2.5 Optical Black Carbon

In addition to gravimetric weighing, the AIRLIFT can be used to automate chemical composition analysis of samples. Aerosol composition can provide valuable information on the source and health/climate implications of the particles sampled onto filters. Although many potential analysis techniques could be streamlined using the AIRLIFT, the first technique added to supplement gravimetric mass was a method of determining black carbon. Black carbon was selected as it was of particular interest to the research team.

Black carbon (BC) is a component of particulate matter generated during incomplete combustion of carbonaceous fuels. Black carbon has been linked to adverse health (Garland et al., 2017; Kim et al., 2004; Lim et al., 2013) and climate effects (Bond and Sun, 2005; Bond et al., 2013) and is therefore of interest to those who study and monitor air quality. A common approach to quantifying BC on filters is to measure the transmission and/or absorption of light (Ahmed et al., 2009; Hansen et al., 1984), often at a wavelength of 880 nm. Although there are known limitations to an optical transmission approach (Babich et al., 2000; Horvath, 1993; Kirchstetter et al., 2004), the measurement's non-destructive nature makes the approach attractive. Optical methods for estimating black carbon are based upon calculating light attenuation (ATN) from a set of differential measurements, as shown in Eq. (1):

Equation 1: Calculation of attenuation of light when passing through a filter and black carbon mass concentration

(1) $$ATN=100\times \text{ln}\left(\frac{I_{0}}{I}\right)\;BC=\frac{ATN}{MAC}$$

where $I_{0}$ is the intensity of light passing through a clean (unused) filter, I is the intensity of light passing through a filter laden with PM, and MAC is the mass-absorption cross-section. The mass of accumulated black carbon, typically reported in units of μg cm^−2^ of active filter surface, is calculated by applying the mass-absorption cross-section for BC on the filter; these values tend to be specific for a given filter type and source (Gundel et al., 1984; Presler-Jur et al., 2017). Although several commercially-available instruments can reliably characterize black carbon using the optical transmission approach, we developed a custom system for integration into the AIRLIFT. This system includes a tungsten halogen laser source (HL-2000-LL, OceanOptics, Cincinnati, OH, USA) and wide-band spectrometer (FLAME-S-VIS-NIR, OceanOptics, Cincinnati, OH, USA) connected through fiber-optic cables. The laser source and spectrometer can emit and detect 260–2000 nm and 350–1000 nm light, respectively. The AIRLIFT optical system uses 880 nm light to quantify BC, but additional wavelengths could be analyzed in the future to expand the AIRLIFTs particle characterization capabilities (Lawless et al., 2004).

### 2.6 Filter Tracking

Each filter is assigned a unique quick response (QR) code. The QR code is typically associated with a filter by placing a sticker on either a filter storage dish or a sampling cartridge, Fig. 2-left. Before every measurement, the AIRLIFT moves the QR code under a scanner, which reads the filter ID and ties that ID with the subsequent measurement.

The AIRLIFT also has an option for taking a photo of each filter during the weighing process. These photos provide traceability information for each sample. The images have sufficient resolution to look for filter defects or uneven particle deposition. Images of the filters are collected using both a USB webcam (1080P Zoopod) and a USB digital microscope (USB2-MICRO-250X, Plugable, Redmond, WA, USA). The webcam is used to record images of the filter while being weighed and the digital microscope for a more detailed analysis of a filter.

### 2.7 Environmental Control

Variations in environmental conditions when weighing filters can change the mass of filters and the performance of the balance itself (Bogen et al., 2011; Charell and Hawley, 1981; Su et al., 2008). U.S. EPA weighing protocols require measurements to be taken at temperatures between 20–23°C with less than 2°C variability and 30–40% relative humidity (RH) with less than 5% variability for at least 24 hours before the filters are weighed (Code of Federal Regulations, 2006). Maintaining consistent temperature and humidity conditions is important when weighing filters. Changes in temperature and RH while measuring a filter can introduce measurement bias (Mettler Toledo, 2016); differing humidity conditions between when a filter is pre- and post-weighed can introduce error due to different amounts of water being absorbed into the filters during conditioning, equilibration, and weighing (Brown et al., 2006).

The AIRLIFT enclosure includes a flow-through humidification and air filtration system. The humidification chamber is a plastic NEMA enclosure filled with powdered magnesium chloride mixed with deionized water to form a saturated solution. A saturated magnesium chloride solution was chosen because it will maintain RH at approximately 35% within the AIRLIFT under typical ambient conditions (Lu and Chen, 2007; Rockland, 1960) (Wexler and Hasegawa, 1954). A fan (SEAFLO, Fujian, China) circulates air through the NEMA enclosure, across the saturated salt solution, and through a high-efficiency particle air filter (HEPA, Air Filters Inc, Houston, TX, USA). The filter used has a high surface area to reduce pressure drop and allow the filter to be used for an extended period of time before replacement. The filter is replaced as part of annual maintenance on the system. Ambient relative humidity conditions in Northern Colorado are typically < 30% RH; thus, moisture is generally released from the salt solution to maintain the target level. The humidification system typically needs to be refilled once or twice a month, depending on ambient conditions and AIRLIFT usage. Fortunately, the room in which we installed the AIRLIFT is already maintained within the required temperature range; therefore, the AIRLIFT enclosure does not require additional temperature regulation.

Temperature and humidity monitors were incorporated into the LabVIEW control system to monitor conditions within the AIRLIFT (temperature: SCASS-125G-6, Omega Engineering Inc., Norwalk, CT USA; RH: HX94BC, Omega Engineering Inc, Norwalk, CT, USA). The AIRLIFT includes a real-time particulate matter sensor (SPS30, Sensirion, Staefa, Switzerland) to monitor background pollution levels to minimize the risk of filter contamination. Although low-cost real-time PM monitors have known limitations (Tryner et al., 2020; Wang et al., 2015), the SPS30 is sufficient for ensuring that extensive infiltration of particles into the AIRLIFT is not occurring. Temperature, RH, and background PM levels are recorded every 30-minutes when the robot is idle and simultaneously with each gravimetric measurement.

### 2.8 Evaluation of AIRLIFT Performance

#### 2.8.1 Gravimetric analysis

We evaluated vibration control efficacy (i.e., marble table and epoxy block) by using the built-in microbalance stability readings to determine the typical time required to get a stable reading. The stability status of the microbalance was recorded every two seconds for a 72-hour period. We then determined the typical time between two consecutive stable readings. The time between stable readings can be used to estimate how long the system would need to wait before a reliable measurement could be collected. Measurement precision was quantified through repeated measures of polytetrafluoroethylene (PTFE) membrane filters (PT25P, Measurement Technology Laboratories, Minneapolis, MN, USA). Repeated measurements of five filters were collected over 35 days. For each weighing session, three measurements for each filter were collected. We determined the average mass change for each filter from day one (long-term stability) and the range in measurements recorded for each filter on each subsequent measurement day (short-term stability). Atypical performance metric for gravimetric analysis is the limit of detection (LOD), which can be calculated as three times the standard deviation of “blank” filter mass change (MacDougall and Crummett, 1980). We calculated LODs at the daily, weekly, and approximate monthly level based on this repeated-measures experiment.

#### 2.8.2 Black carbon analysis

The Magee Scientific SootScan (OT21 Magee Scientific, Berkeley, CA, USA) is one of the most commonly-used commercial instruments for optical analysis of black carbon. We evaluated the AIRLIFT optical BC system's performance in terms of agreement with the SootScan and repeatability of replicate measurements. We determined attenuation for eight neutral density disks, twenty-two 37-mm Teflon membrane filters (PT37P, MTL Corp, Minneapolis, MN, USA), and twenty-two 37-mm Teflon coated glass-fiber filters (Emfab, Pall Corporation, Port Washington, NY, USA). We loaded filters with PM emitted from burning wood and diesel emissions; each filter was measured using the SootScan and the AIRLIFT optical BC system before PM loading and again after loading. Pre-loading measurements were collected to account for filter material variability (Presler-Jur et al., 2017). The attenuation of ten membrane and ten glass-fiber filters was measured five times after loading to evaluate measurement repeatability.

#### 2.8.3 Environmental control

The environmental control system's efficiency was evaluated by looking at trends in temperature, humidity, and background PM levels over time. Temperature and humidity were compared against EPA requirements. Although there are not strict EPA guidelines for control of background air quality during gravimetric analysis, monitoring of enclosure air quality (and use of an internal HEPA filter) seemed an important quality metric for the AIRLIFT.

## 3 RESULTS AND DISCUSSION

### 3.1 Robotic Filter Handling and Storage

Commercial automated weighing systems typically use 2-axis cartesian robots; the AIRLIFT departs from conventional designs by using a 6-axis robot arm. Cartesian robots generally are easy to program and can provide advantages when aligning components. However, the fixed motion range of Cartesian robots can be limiting if seeking to expand or modify a system. Additionally, Cartesian robot movement often comes from rack and pinion or gear and belt designs which raise concerns for filter contamination because debris and small particles can form as these moving parts abrade over time (Kohara Gear Industry Co., 2015). The use of external seals may be required to mitigate this impact. While the multi-axis robot arm also includes gears and moving parts, all the wearing parts are sealed within the robot's joints, reducing the potential for air contamination. A multi-axis arm also allows flexibility in component placement within the AIRLIFT and facilitates expansion of the system to include other measurement techniques.

### 3.2 Gravimetric Measurements

**Fig. 4.**
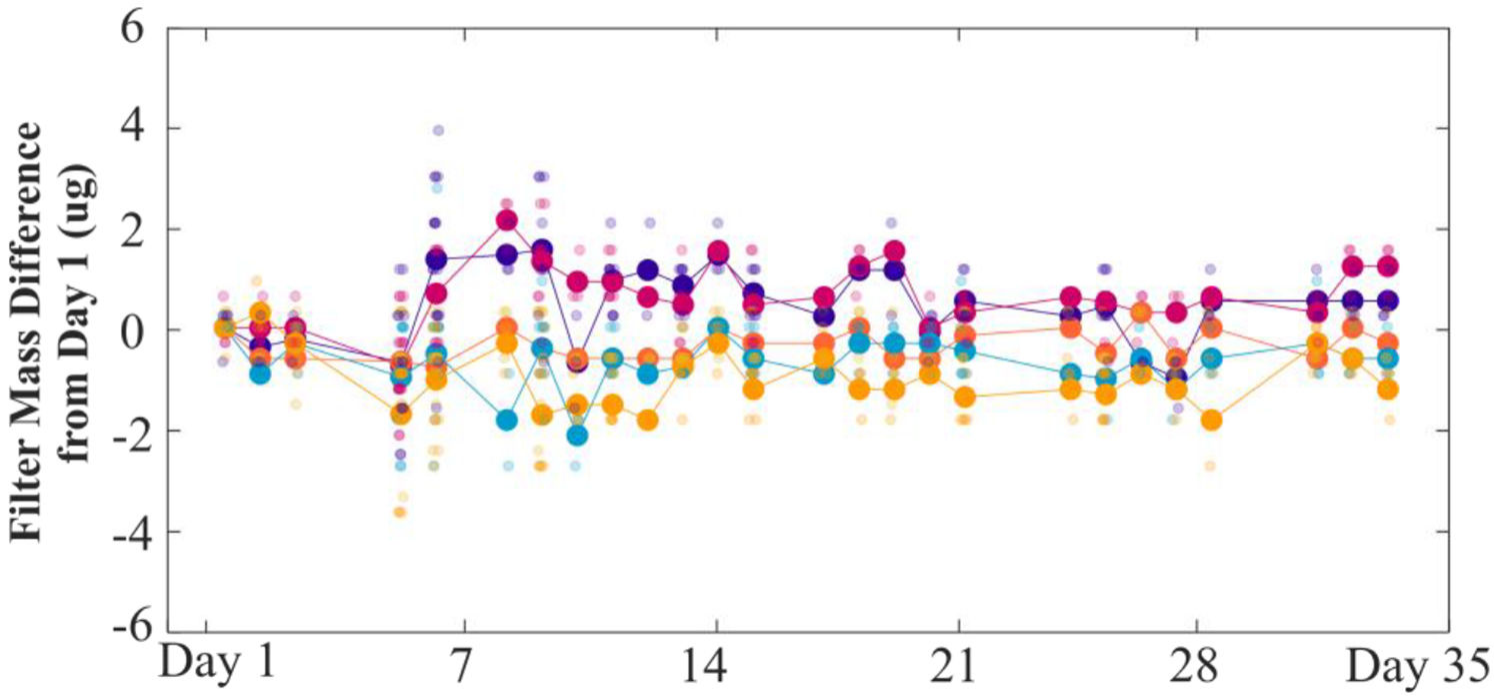
Repeated measures of five filters over a period of 35 days. The five different marker colors represent the five different filters. Each large circle represents the average of three measurement replicates per day and is normalized to the average mass of that filter on day 1. The smaller circles indicate the individual measurement replicates.

Results for gravimetric measurement stability are shown in Fig. 4. The change in filter mass, for each of five filters, was compared to the average mass for each respective filter on day 1. The average absolute mass change from day 1 (i.e., mass difference on any given day compared to day 1) was 0.8 μg ± 0.5 μg (N = 125); substantially less than the 15 μg requirement specified by the U.S. EPA. Across the 35 days of repeated measurements, each filter’s average mass change remained within 4 μg of the mass on day 1 with no discernable systematic drift over time.

**Fig. 5.**
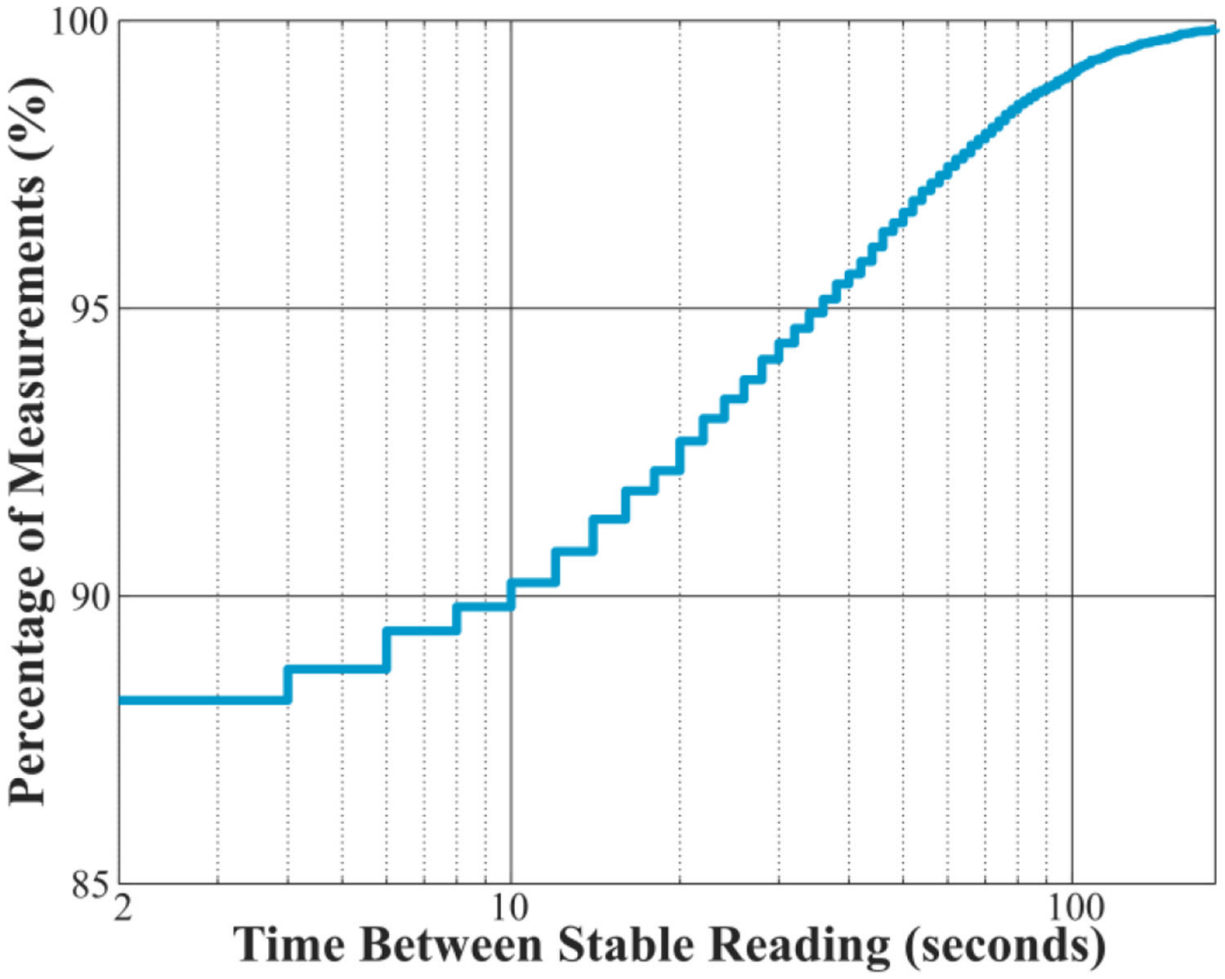
Cumulative distribution function plot of time between stable readings.

The precision of a microbalance degrades when operated in a room with excessive vibration. We evaluated balance sensitivity to external conditions by recording stability status every two seconds for 72 hours. The area near the AIRLIFT had individuals completing routine laboratory activities for approximately half of those 72 hours. The microbalance reported a stable reading 90% of the time within 10 seconds for typical work hours and 97% of the time within 60 seconds, Fig. 5. By programmatically waiting for a stable balance reading, the AIRLIFT can collect quality measurements even in a room used for other activities. It should be noted, although design elements were included to help mitigate vibrations (e.g., the marble table), selection of where to place the microbalance has a large influence on balance performance. The AIRLIFT only includes passive vibration control. Therefore, the same measurement configuration in a different location could result in different (better or worse) performance.

The AIRLIFT detection limit was estimated by looking at the change in filter mass across subsequent measurements. Although LODs are typically measured using filter blanks (i.e., filters with no loading), the most important metric is the mass change of a filter over time. We estimated LOD by calculating three times the standard deviation of mass change for the five filters across time. The average LOD was 2.7 μg (max: 5.1 μg; min: 0.7 μg), with no discernable systematic drift over time. The low LOD indicates that the AIRLIFT would be appropriate for measuring filters used in studies with relatively low total particulate matter loading, assuming gravimetrically stable filters and proper handling protocols are used during filter collection. It should be recognized that the LOD presented above would represent a best case scenario. Detection limits from field studies will be influenced by many other considerations such as handling and transportation of filters.

### 3.3 Optical Black Carbon

**Fig. 6.**
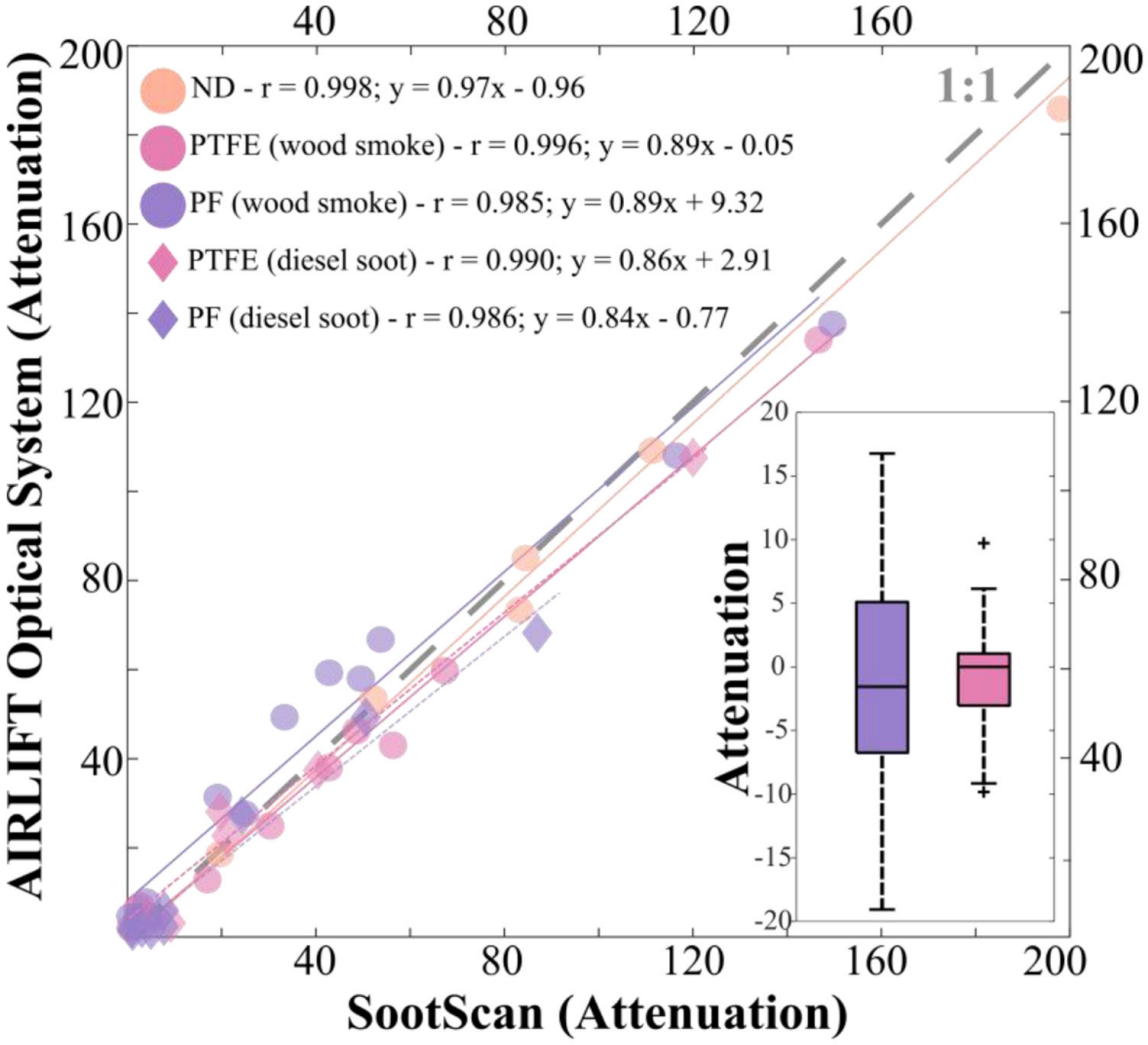
Attenuation determined by Magee Scientific SootScan and AIRLIFT Optical BC System. Eight neutral density (ND) disks and twelve of each of 37-mm MTL PTFE filters (PTFE) and 37-mm PallFlex Emfab filters (PF) were evaluated. MTL wood smoke: n = 12, MTL diesel soot: n = 10, Emfab wood smoke: n = 12, Emfab diesel soot: n = 10, Neutral density disk: n = 8. (Insert) Variation in repeated attenuation measurements. Plus symbols represent outlier points.

Black carbon quantification accuracy was evaluated by comparing loaded filter attenuation as determined by the AIRLIFT optical BC system to that of the Magee Scientific SootScan. A total of 32 comparisons were made: 8 neutral density glass disks, 22 Teflon membrane filters, and 22 Teflon coated glass-fiber filters (N total = 52). Each filter was evaluated before and after loading with PM to account for potential inter-filter variability. Method agreement was quantified in terms of a Pearson’s coefficient (r) and the slope/y-offset of a least-squares fit regression line. A strong linear relationship was found for the three material types tested, all with 0.84 > slopes > 0.98 (Fig. 6).

Measurement precision for the AIRLIFT optical BC system was quantified by collecting five repeated measures of 20 filters (ten Teflon membrane and ten Teflon coated glass-fiber) (N total = 100). Each subsequent measurement was normalized to the attenuation calculated for the first measurement. Repeat measurement variability has been represented as boxplots, Fig. 1-Insert, with the box bounds representing the 25^th^ and 75^th^ percentiles. Both filter media tested had median variability in attenuation of less than 2 (absolute). The interquartile ranges for the MTL and Emfab filters were 4.1 and 11.8, respectively.

### 3.4 Environmental Control

**Fig. 7.**
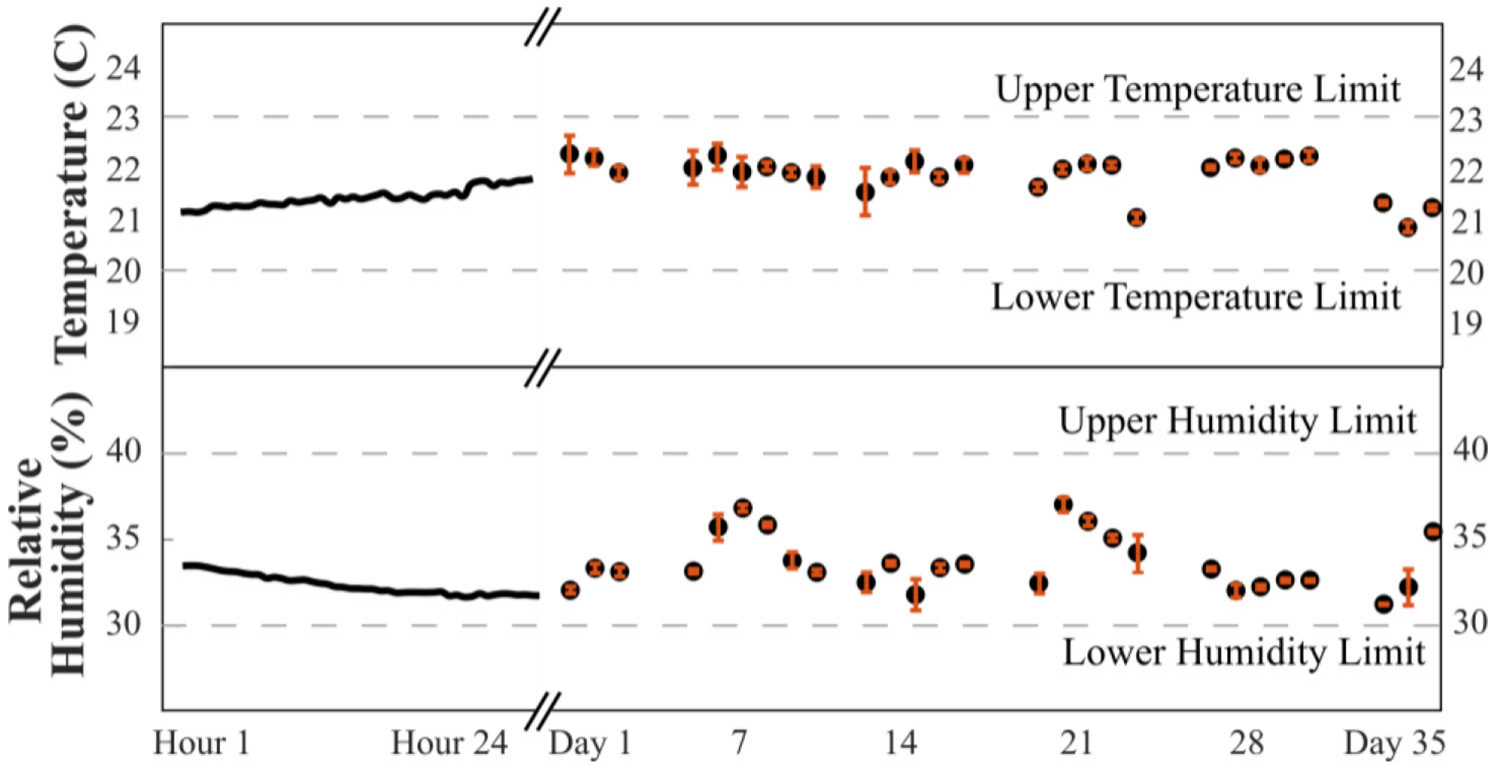
Mean temperature and humidity conditions within the AIRLIFT. Temperature and humidity conditions measured every 30 minutes for 24-hours and as well as anytime filters were equilibrating in the AIRLIFT over 35 days. Gaps in the time series are days the system was in "idle mode" and no filters were equilibrating. Error bars represent ±1 standard deviation of measurements collected on that day.

We evaluated short-term and long-term AIRLIFT temperature and humidity stability. Measured temperature and humidity were within EPA specifications for both 24 hour periods and periods spanning multiple weeks (Fig. 7). The largest variations in relativity humidity corresponded to the days after water addition to the salt solution (Fig. 7). Decreasing the amount of water added to the salt solution on any given day but increasing the frequency of addition could reduce variability. Outdoor temperatures and relative humidity conditions varied from −17 to 23°C and 0 to 100%, respectively, during the testing window (March 11–April 15, 2020).

The environmental control and filtration system has a nominal flow rate of approximately 300 L min^−1^ when running; the blower is off while a filter is on the microbalance to minimize air currents within the AIRLIFT. While the blower is on, this flow rate equates to 4.6 air exchanges per hour through the HEPA filter. The average PM_2.5_ concentration, as reported by the SPS30, over a 72-hour period was 1.3 ± 0.7 μg. We try to minimize the time the AIRLIFT doors are open, typically to load or unload filters, to reduce the risk of external particles entering the system.

### 3.5 Usability

We designed the AIRLIFT to collect quality gravimetric filter data and minimize the labor burden on laboratory personnel. We estimate that each filter requires 60 seconds of personnel time. This time includes applying a QR code, loading the filter on a tray, placing the tray in the AIRLIFT, initiating the program, and unloading the filter after weighing. The time required per filter varies slightly with the number of filters being prepared (e.g., the time to initiate the program does not change with the number of filters to be analyzed), but this variation is small. Although external factors such as vibration levels influence the time required to collect a measurement, the AIRLIFT can typically weigh a filter in less than 4.5 minutes. With an estimated total time of 5.5 minutes, the AIRLIFT could theoretically analyze 261 filters per day. We can also quantify AIRLIFT usability in terms of personnel time saved. We estimate the AIRLIFT takes less than 25% of the personnel time required for manual weighing of filters; this is based upon comparing the approximately 60 seconds total to prepare the filter and load/unload the filter from the AIRLIFT to the approximately 6 minutes to manually weigh a filter. This reduction in labor is likely conservative as the automation of the process also reduces the need for reweighing filters due to human error.

The AIRLIFT has allowed us to decrease the personnel time dedicated to filter preparation. As a result, it allowed us to increase the number of filters collected for our air quality studies. The AIRLIFT has been used to perform nearly 80,000 mass measurements between May 2018 and October 2020, Fig. 8. The “on-demand” ability of the AIRLIFT has allowed for large fluctuations in the number of filters processed per month without massive disruptions in personnel schedules. The AIRLIFT has also allowed for research efforts to continue during periods of uncertainty or limited personnel availability such as the COVID19 pandemic.

## 4 CONCLUSIONS

We designed a high-throughput, robotic system to facilitate analysis of gravimetric filters at large volumes. The AIRLIFT is comprised of readily-available equipment and is assembled using basic construction techniques. We designed the AIRLIFT to measure gravimetric filters per U.S. EPA guidelines while being configurable for additional non-destructive filter analysis techniques.

Our results show that the AIRLIFT meets the temperature and humidity conditions required by the U.S. EPA filter analysis protocol and achieves the measurement repeatability necessary for air quality related studies. An automated approach, such as the AIRLIFT, would allow research groups to allocate valuable personnel time to scientific endeavors instead of the tedious time-consuming process of manually weighing filters.

## Supplementary Material

Supplemental Material

## Figures and Tables

**Fig. 8.**
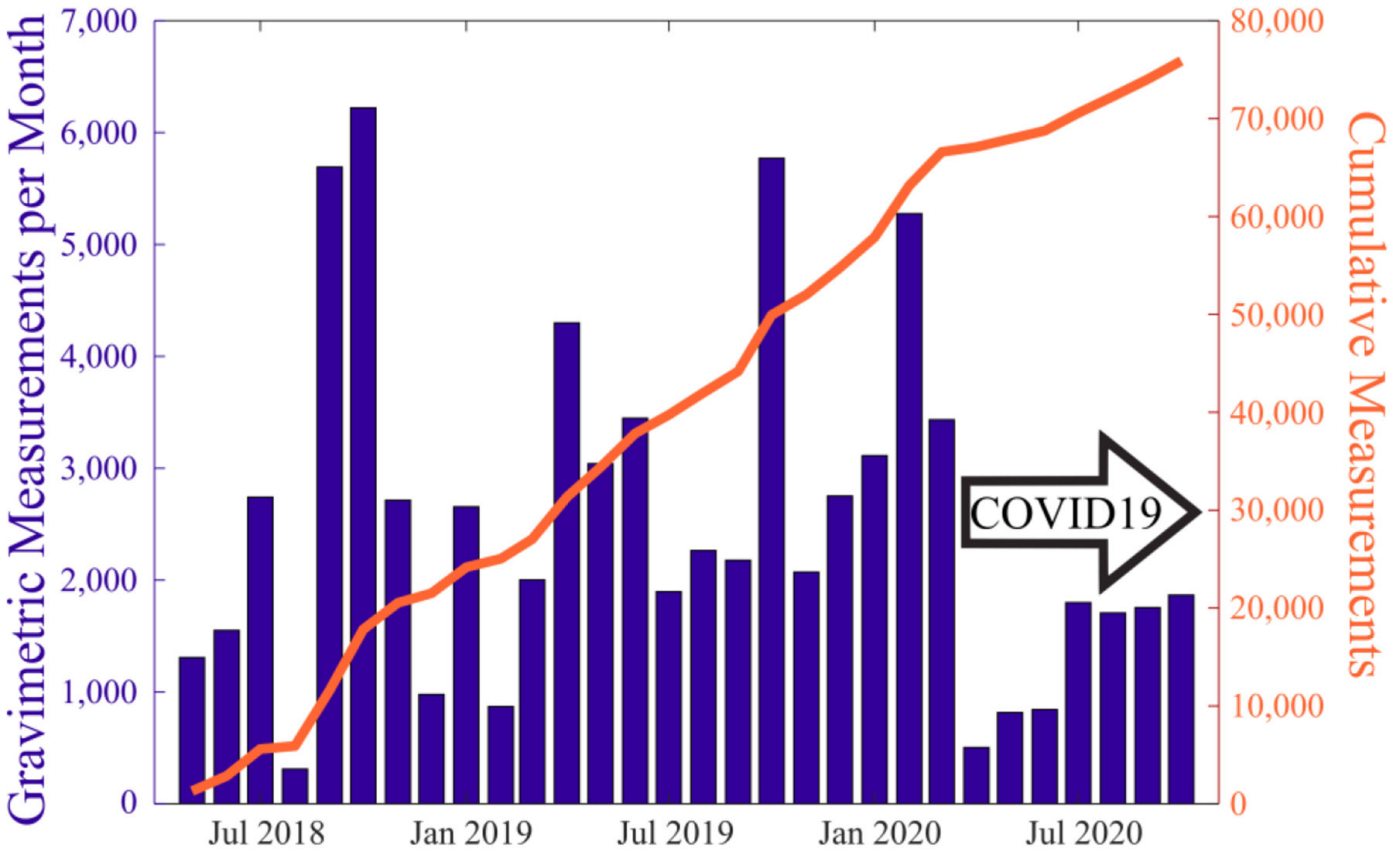
Number of gravimetric measurements collected per month between May 2018 and October 2020 (left axis) and the cumulative number of mass measurements collected in that period (right axis).
