# Supplementary material for: A High-throughput, Robotic System for Analysis of Aerosol Sampling Filters
Source: Aerosol Air Qual Res. Author manuscript; Available in PMC 2024 Jan 8. (PMC10772922; doi:10.4209/aaqr.210037)
Supplement: Supplemental Material [file NIHMS1951970-supplement-Supplemental_Material.pdf]

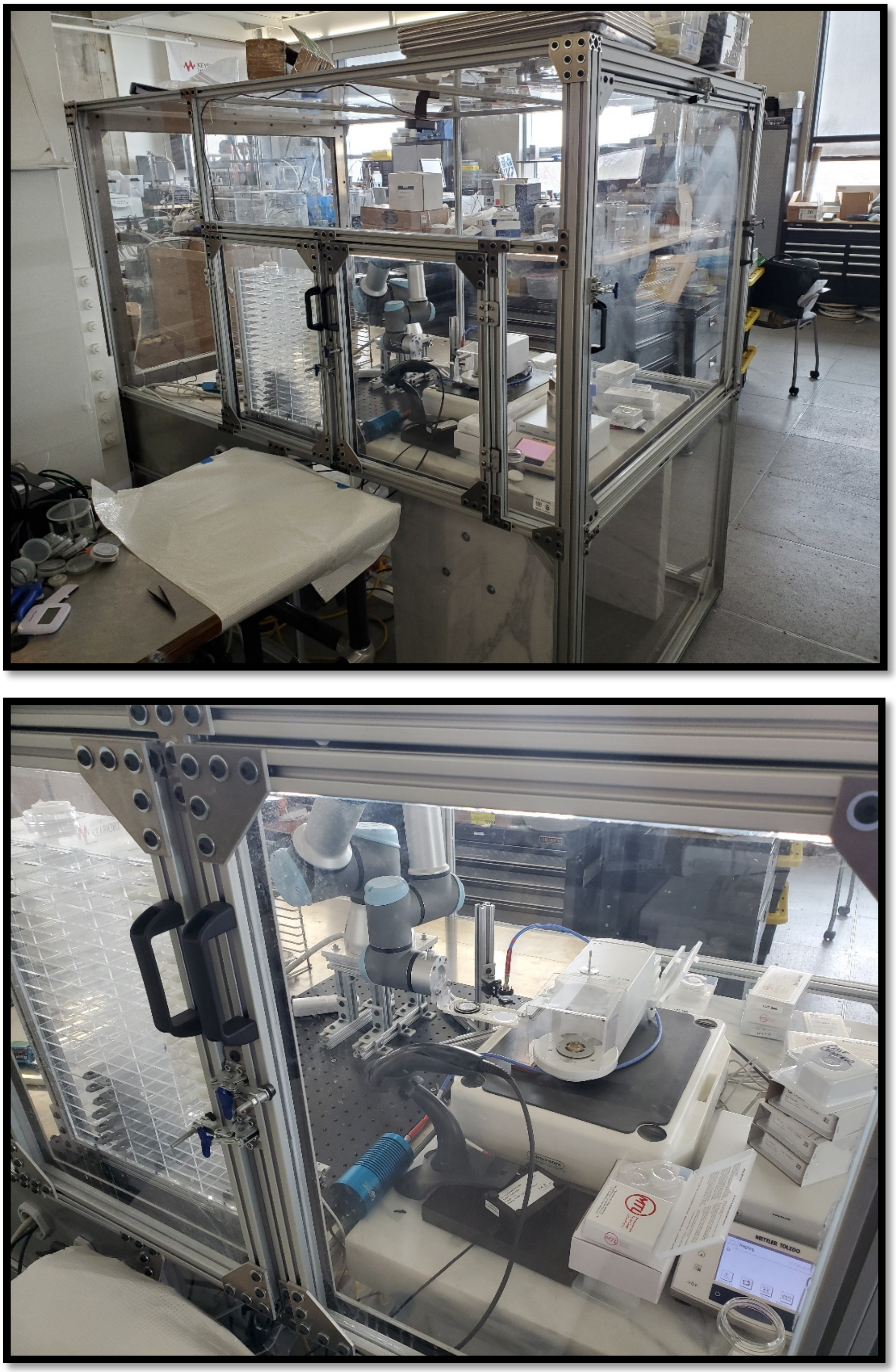

Figure S1: Example photos of the AIRLIFT weighing system. (Top) Image of AIRLIFT system in the Advanced Aerosol Laboratory at Colorado State University. (Bottom) Image showing the robotic arm about to neutralize a filter using the radiation source and then deposit the filter on the microbalance. The filter storage rack can be seen in the left edge of the photo.

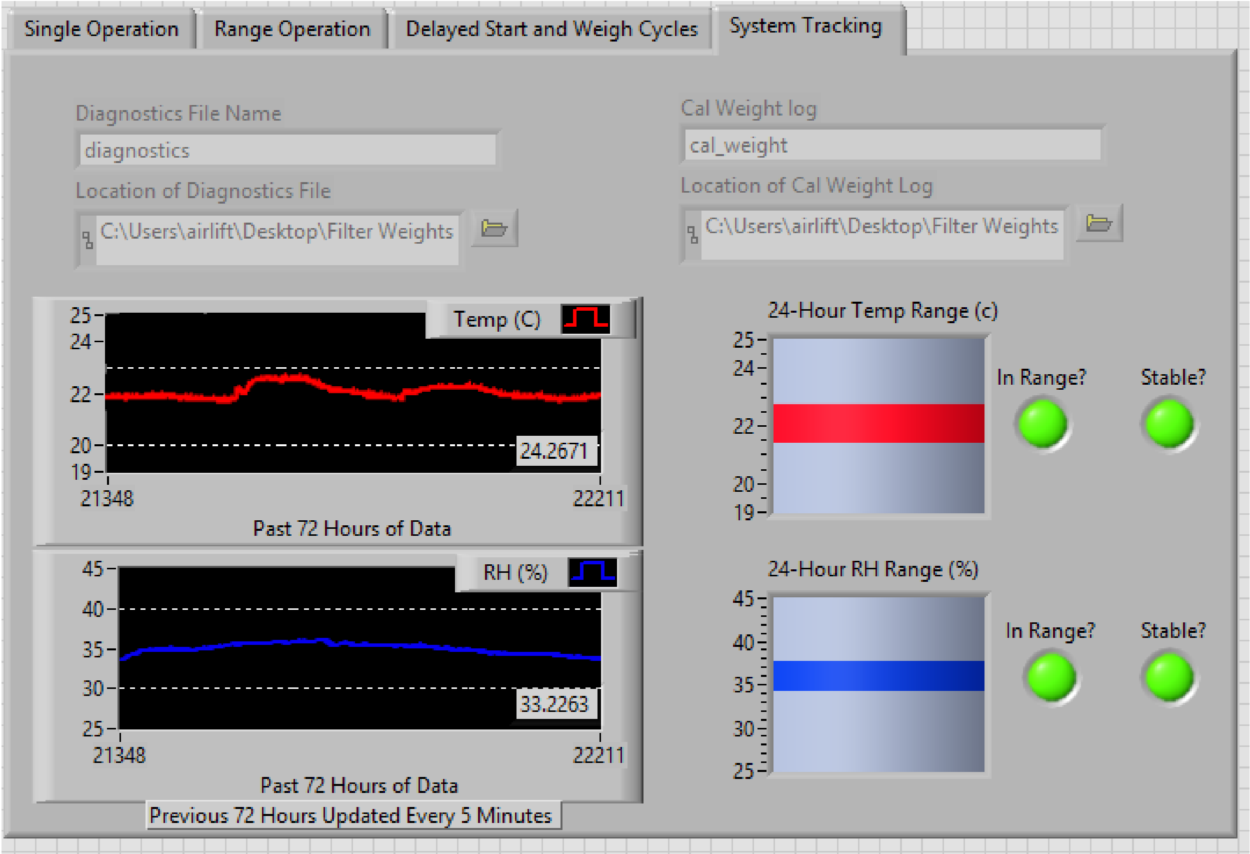

Figure S2: Examples of historical temperature and humidity conditions within the weighing system and examples of some of the quality control information which is presented to AIRLIFT operators.

Table S1: Major AIRLIFT components with brief description and approximate price at the time of purchase.

Component	Model	Description	Approximate Price (USD)
Microbalance	Mettler Toledo XS3DU	• 1 ug precision<br>• 1 ug repeatability<br>• 5.1 g max capacity	$26,000
6-axis Robot	Universal Robots UR3	• 6 degrees of freedom<br>• 0.1 mm repeatability<br>• 3 kg capacity<br>• 500 mm reach	$20,000

Enclosure	Custom	• 4 m^3 volume<br>• Acrylic and extruded aluminum construction<br>• Integrated saturated salt humidity control<br>• 100 filters/batch measurement capacity<br>• >1,000 filter equilibration capacity	$5,000
Neutralizer	Po 210	• Alpha emitter<br>• 13-19 mm distance from filter for optimal neutralizing	$200
Data Acquisition System	National Instrument cRio 9066	• Embedded control with real-time processing capabilities<br>• Equipped with analog and digital inputs/outputs	$6,000
